# IUC24425-82 Nanosensors for early-stage detection of circulating tumor cell biomarkers in prostate cancer diagnosis: a systematic review of published evidence

**DOI:** 10.1093/oncolo/oyaf248.009

**Published:** 2025-08-29

**Authors:** S Basu, J K Tan, S Adeleke, S Boussios, A Ghose

**Affiliations:** University College London Cancer Institute, London, United Kingdom; University of Manchester, Manchester, United Kingdom; Cancer Centre at Guy’s, Guy’s and St Thomas’ NHS Foundation Trust, London, United Kingdom; Medical Oncology, University Hospital of Ioannina, Ioannina, Greece; Barts Cancer Centre, Barts Health NHS Trust, London, United Kingdom

## Abstract

**Background:**

Prostate cancer (PCa) remains the most commonly diagnosed malignancy among men worldwide, with stage IV metastatic PCa exhibiting a 5-year survival rate of 27%. The current standard for diagnosis employs prostate-specific antigen (PSA) testing, lacks specificity, and suffers from a high limit of detection (LOD), leading to misdiagnosis and false positives. Liquid biopsies, particularly circulating tumor cells (CTCs), offer a promising alternative, providing rapid and more accurate prognostic diagnosis. Nanosensors, which are highly sensitive and non-invasive, are a patient-friendly alternative. Our objective was to critically evaluate the applications of 4 nanosensors-gold nanoparticles (AuNPs), quantum dots (QDs), magnetic nanoparticles (MNPs), and graphene/GO-based nanosensors for early-stage CTC detection in PCa diagnosis.

**Methods:**

A systematic search for primary literature was conducted on PubMed, which included *(“Prostate Cancer” AND “CTC” AND “Biomarker”) AND (Nanosensor AND Nanotechnology) AND (Early Detection).* Outcomes include LOD, sensitivity, biocompatibility, and range. The search strategy was filtered to include studies published in English between 2014 and 2024. Abstracts and titles were screened to yield 4 primary research papers and 10 systematic reviews for evaluation.

**Results:**

Upon analysis, MNPs demonstrated the highest sensitivity with a LOD of 0.001 ng/mL. QDs also performed well, with a LOD of 0.009 ng/mL and the ability to detect both free and complex PSA within a short assay time. AuNPs followed with a moderate LOD of 0.02 ng/mL and good biocompatibility, though limitations in stability, reproducibility, and cost. Graphene sensors exhibited a broader LOD range (0.07-0.2 ng/mL), good surface area-to-volume ratio, and miniaturization potential, but faced issues with scalability and cost. Compared to the conventional PSA test, all nanosensors showed improved sensitivity and specificity. However, individual drawbacks, such as cytotoxicity (QDs), aggregation in biological fluids (MNPs), and photochemical disturbances, impacted their translational potential.

**Conclusions:**

MNPs show great promise for early prostate cancer detection due to their superparamagnetism, stability, and biocompatibility. Nanosensors outperform conventional PSA tests but lack standardized protocols and sufficient data. Prospectively, more research needs to be conducted to derive comparable data and improve biosafety, specificity, and signal amplification for these nanosensors.

